# Vasoactive intestinal peptide alleviates osteoarthritis effectively via inhibiting NF-κB signaling pathway

**DOI:** 10.1186/s12929-018-0410-z

**Published:** 2018-03-14

**Authors:** Yaozhong Liang, Shu Chen, Yuhao Yang, Chunhai Lan, Guowei Zhang, Zhisheng Ji, Hongsheng Lin

**Affiliations:** 1 Department of Orthopedics, The First Affiliated Hospital of Jinan University, Guangzhou, 510630 China; 2 Department of gynaecology and obstetrics, The First Affiliated Hospital of Jinan University, Guangzhou, 510630 China

**Keywords:** Vasoactive intestinal peptide, Osteoarthritis, Synoviocytes, NF-κB signaling

## Abstract

**Background:**

To investigate the treatment effect of vasoactive intestinal peptide (VIP) on osteoarthritis (OA) and the relative mechanism.

**Method:**

The OA model on the SD rat knee was established using the modified Hulth method, and the recombinant pcDNA3.1+/VIP plasmid was constructed. One month after the plasmids VIP were injected intra-articularly into the right knee joint of OA and sham-operated rats, the pathological changes of the OA knee joint were observed by Hematoxylin-eosin (HE) and Safranin O/fast green staining. The levels of VIP and serum inflammatory cytokines (TNF-α, IL-2 and IL-4) were measured by ELISA kits. Meanwhile, synoviocytes isolated from OA rat and sham-operated rat were cultured in vitro, and transfected with the VIP plasmid. The proliferation of synoviocytes was determined using BrdU kits. The protein expressions of TNF-α, IL-2, CollagenII, osteoprotegerin (OPG), matrix-degrading enzymes (MMP-13, ADAMTS-5), and the related protein of NF-κB signaling pathway (phosphorylated p65, phosphorylated IκBα) were evaluated by western blot.

**Results:**

The VIP plasmid could effectively improve the pathological state of the OA rats knee joint, significantly decrease the levels of serum TNF-α and IL-2, and clearly increase the levels of VIP and serum IL-4. At the same time, after the OA synoviocytes were treated with the VIP plasmid, the proliferation ability of OA synoviocytes was reduced, the protein expressions of Collagen II and OPG were remarkably up-regulated, and the protein expressions of TNF-α, IL-2, MMP-13 and ADAMTS-5 were significantly down-regulated. In addition, the p-p65 expression decreased and p-IκBα expression increased.

**Conclusion:**

Osteoarthritis was effectively treated by VIP via inhibiting the NF-κB signaling pathway.

## Background

Osteoarthritis (OA) is a common chronic degenerative and disabling joint disease, that has a high incidence in the elderly, and seriously affects people’s health and quality of life [1]. Today, OA is considered as a major public health problem worldwide, and the prevalence of OA increases year after year. By 2030, it is predicted that in the United States 67 million people will be diagnosed with OA [2]. Clinically, the disease is characterized by persistent joint pain and joint disorder, accompanied by a gradual loss of articular cartilage, osteophyte formation, subchondral bone sclerosis, synovium inflammation, the proliferation of synoviocytes, and joint function [3, 4]. It was reported that the occurrence of OA is associated with a variety of factors, such as age, trauma, and genetic factors, but no significant racial and geographical differences have been described [5]. However, until now, the exact causes and pathogenesis of OA have not been entirely clear, and in the clinic, there is no effective therapeutic strategy to treat OA. Therefore, in recent years, clarifying the pathogenesis of OA and finding an effective method for its treatment have gained more and more attention.

Vasoactive intestinal peptide (VIP) is an immunologically active neuropeptide, that has anti-inflammatory and immune-regulating effects. It has been reported that VIP can relieve and treat experimental arthritis [6, 7]. Moreover, Jiang et al. [8] demonstrated that VIP is a potential indicator of severity of OA. However, due to the short half-life and fast metabolism of VIP in vivo, and because the long-term injection of VIP will cause immune suppression and gastrointestinal disorders, the clinical application of VIP is greatly restricted [9]. Recently, gene therapy has been developed to effectively avoid these side effects.

Therefore, in the present study, the VIP recombinant plasmid was constructed, and the OA model rat was established using modified Hulth method. Then, the effect of VIP recombinant plasmid on OA model rat was investigated in vivo*.* In addition, an OA synoviocyte model was established, and the cultured OA synoviocytes with or without VIP recombinant plasmid were evaluated in vitro. Furthermore, the underlying mechanism of VIP was explored.

## Materials and methods

### Materials

VIP primer was synthesized by Sangon Biotech (Shanghai) Co., Ltd. (Shanghai, China). EcoR I and Hind III were obtained from MBIFermentas (Vilnius, Lithuania). Cell culture medium and supplements were purchased from Gibco (Mountain View, CA, USA). pcDNA3.1, Lipofectamine 2000 and TRizol were obtained from Invitrogen Corp. (Carlsbad, California, USA). The VIP ELISA assay kit was purchased from Solarbio (Shanghai, China). TNF-αELISA assay kit, IL-2 ELISA assay kit, IL-4 ELISA assay kit and the 5-bromo-2-deoxyuridine (BrdU) cell proliferation assay kits were purchased from Merck MilliporeCorp (Darmstadt, Hesse, Germany). Antibodies of VIP, TNF-α, IL-2, CollagenII, OPG, MMP-13 and ADAMTS-5 were purchased from Abcam (Cambridge, MA, USA). Antibodies of Vimentin, p65, p-p65, IκBα, p-IκBα, β-actin and IgG were purchased from Cell Signaling Technology. Inc. (Danvers, MA, USA).

### Methods

#### Construction of VIP recombinant plasmid

VIP recombinant plasmid was constructed according to a previously reported method [10, 11]. Briefly, mouse thymocytes were taken and total RNA was extracted using TRIzol reagent. Then,1 μg of total RNA was used to clone VIP cDNA by RT-PCR. The primers used were as follows: F-GCCAAGCTTATGGACACCAGAAATAAGGCCCAGCTCCTTGTGCTCCTGACTCTTCTCAGTGAGCTCTTCTCACACTCTGATGCCGTCTTC; R-CGGAATTCTCAATTCAGGATGGAGTTCAG-3. The reaction conditions were as follows: 94 °C 3 min, 94 °C 30 s, 58 °C 30 s, 72 °C 30 s for 5 cycles; 94 °C 30 s, 68 °C 30 s, 72 °C 30 s for 25 cycles, and 72 °C extension for 10 min. The PCR products were identified by electrophoresis in a 2% agarose gel. PCR products were digested by EcoR I and Hind III, and the target fragments were then recovered and directed to the eukaryotic expression vector pcDNA3.1 treated with the same double digestion and recovered with T_4_ DNA ligase. Then, DH5α bacteria were transformed, and plasmid was extracted and enzyme digested. Subsequently, the products were identified by electrophoresis and sequencing.

#### Establishment of OA rat model

Fifty-six SD male rats (5 weeks old, 250–280 g in weight) were purchased from Guangdong Pharmaceutical University. Rats had access to water and food ad libitum, and were maintained under a 12-h light/dark cycle, at a controlled room temperature (25 ± 1 °C). The animal experimental protocols and the use of chloral hydrate as a surgical anesthetic were approved by the guidelines of Animal Care and Use Committee of Guangdong Pharmaceutical University. All experimental procedures were performed in compliance with the NIH Guide for the Care and Use of Laboratory Animals and National Animal Welfare Law in China.

The OA rat model was established by performing unilateral knee joint surgery using a modified Hulth method as described by Hayami et al. [12]. Twenty-eight rats (5 weeks old, 250–280 g in weight) were randomly selected and anesthetized with 10% chloral hydrate (4 mL/kg, i.p.) according to previous reports [13]. Then, the rats were laid supine on the operating table and received hair removal and povidone-iodine disinfection. The right knee joint cavity of rat was exposed via a medial parapatellar incision. Subsequently, the anterior cruciate ligamentand medial collateral ligament were cut off, and medial meniscus was resected. Then, the incisions were sutured layer by layer. After the surgery, to prevent infection, the rats received 20,000 U penicillin intramuscular injections every day for a week. The remaining twenty-eight SD rats were grouped in the sham surgery group; these rats were treated in the same manner as the OA rat model, but the anterior cruciate ligament and medial collateral ligament were not cut off, and the medial meniscus was not removed. Four weeks after surgery, four rats from the sham-operated group and OA model group were randomly selected for sacrifice to verify by pathological observation whether the OA rat model set up successfully.

#### Grouping and administration

The sham-operated and OA rats were both randomly divided into two groups: sham+control plasmid (A), sham+VIP plasmid (B), OA + control plasmid (C) and OA + VIP plasmid (D). After anesthesia, the rats in groups A and C and groups B and D received intra-articular injection of 15 μL of control plasmid or VIP plasmid (pcDNA3.1+/VIP 30 μg), respectively, into surgical site once a week for 4 consecutive weeks. During the experiment, the rats were allowed free access to food and water, and the state of rats were observed every day to avoid any adverse events. Two hours after the last administration, 5 mL of whole blood was obtained from the abdominal aorta, and centrifuged immediately. Collected the supernatant and placed at 4 °C until measured. The rats were subsequently euthanized by inhalation of carbon dioxide. Additionly, in each group of rats, the right knee joints were taken and the synovial tissue was separated, and then stored at − 20 °C before the assay.

#### Histological examination of knee joint

The tissues of rat knee joints were fixed in 10% paraformaldehyde and decalcified in 20% EDTA solution. Then, the specimens were cut along the sagittal plane, which were then dehydrated in ethanol step by step, vifrificated in xylene and embedded in paraffin. Subsequently, the specimens were cut into 4.0~ 5.0 μm sections, then stained with HE and Safranin O/fast green. Histological changes were observed under a light microscope.

The Osteoarthritis Research Society International (OARSI) Scoring System was used to analyze cartilage degenerative status described previously as follows [14]:The structure was scored on a scale of 0–6, where 0 = surface intact, cartilage morphology intact; 1 = surface intact; 2 = surface discontinuity; 3 = vertical fissures (clefts);4 = erosin; 5 = denudation; 6 = deformation. Histological evaluation was performed by two independent experienced researchers who were blinded to information of the study.

#### Detection of serum VIP and cytokines

The serum VIP and serum cytokine (TNF-α, IL-2 and IL-4) levels in each group were measured using ELISA assay kits according to the manufacturer’s instructions. Optical density was detected at 450 nm through a SpectraMax M5 autoreader.

#### Isolation and culture of synoviocytes

Synoviocytes were isolated using the type II collagenase digestion method. First, Synovial tissues of OA rats and sham rats were taken and minced for digestion with 0.2% collagenase II for 3 h. Then, the solutions were filtered using a 200 mesh sieve to collect the filtrate and were centrifuged at 1200 r/min for 5 min. Subsequently, the supernatant was discarded, and the appropriate amount of DMEM culture medium containing 10% FBS (Gibco) were added. Finally, the cells were cultured in a 37 °C, 5% CO_2_ incubator, and the medium was changed once every 48 h. The growth of the cells was observed under an inverted microscope. When the confluence reached approximately 80%, the cells were passaged. The cells of generation 3 were were used for the following experiments. Cells isolated from sham and OA rats were named as sham and OA synoviocytes, respectively, and they were identified by immunofluorescence staining.

#### Transfection

The synoviocytes were plated at a density of 3 × 10^5^per well on 6-well plate in DMEM containing 10% FBS. When the cells grew to approximately 80% confluence, they were then transfected with 10 nM of VIP plasmid or empty-pcDNA3.1vector (as NC plasmid) by Lipofectamine 2000 reagent for 48 h following the instructions of manufacturer.

#### Cell proliferation assay

Cell proliferation was detected by the BrdU Cell Proliferation assay kit according to the manufacturer’s instructions.

#### Western blotting

The cells were lysed with RIPA and centrifuged. Protein concentrations were detected using the BCA protein assay kit. The protein was fractionated using SDS-PAGE and transferred onto PVDF membrane. Before incubating with antibodies, membranes were first blocked for in 5% skim- milk. Then the membranes were incubated with primary antibodies at 4 °C overnight, followed incubated with HRP-conjugated IgG antibody at room temperature for 1 h. Finally, the protein was visualized using the ECL kit and observed by the GeneGnome machine (Syngene).

#### Statistical analysis

All data are presented as the$\overline{\ x}$±SD and were analyzed using SPSS 13.0 software. Comparisons between groups were processed by one-way analysis of variance, with *P* < 0.05 considered significant.

## Results

### Histological changes

**Fig. 1:**
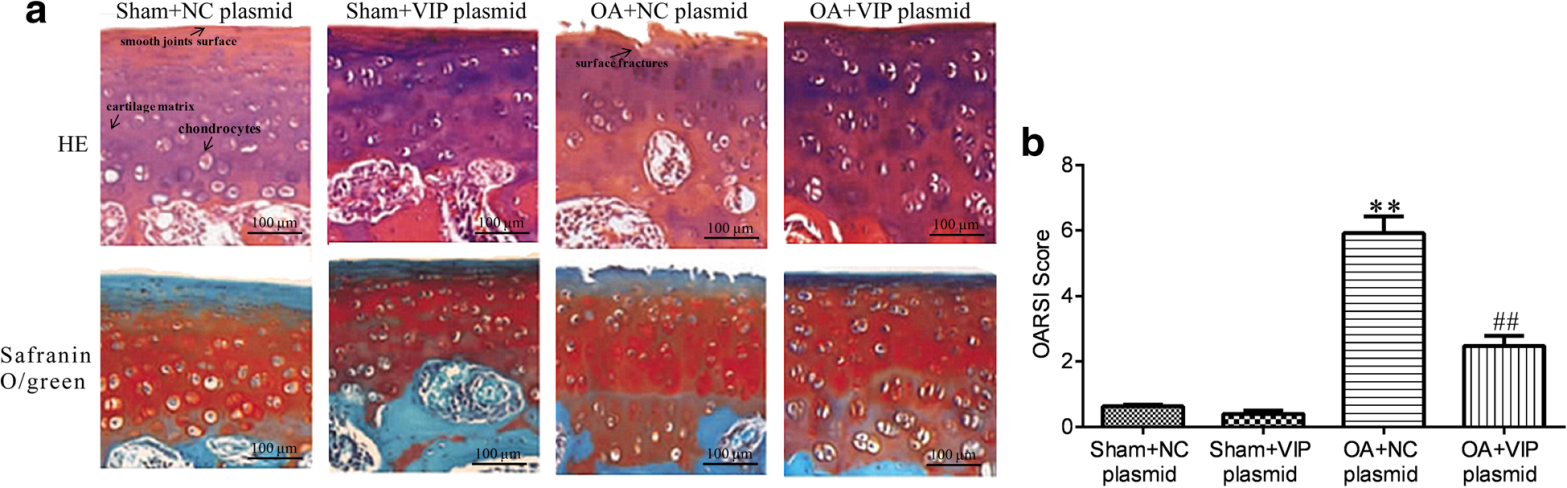
Histopathologic profiles of rat knee joints in each group by hematoxylin and eosin (HE) staining and Safranin O/fast green staining (**a**) and OARSI score (**b**). (Scale bar = 100 μm)

To observe the effect of VIP plasmid on the OA knee joint, HE and Safranin O-fast green staining were performed. As shown in Fig. 1a, there was no significant changes of pathological features between the rats’ knee joint in sham+control and sham+VIP plasmid group, and smooth joints surface, clearly visible cartilage structure, a complete tide line were observed. However, in the OA + control-plasmid group, there were some fractures in the surface, the numbers of chondrocytes notably reduced, cells were diffusely increased, and a large number of cells clusters appeared, which was consistent with the basic pathological changes of osteoarthritis. In contrast, in the OA model rats treated with VIP plasmid, the pathological changes of the rat knee were significantly improved. Consistently, as shown in Fig. 1b, we found that the OARSI score in OA + VIP plasmid group was significantly lower than that in OA + control plasmid group. After conducting statistical analysis, there were significant differences in the OARSI scores after the injections of VIP plasmid compared to OA model rat treated with control plasmid alone (*P* < 0.01).

### Analysis of the serum VIP and serum cytokines

The serum VIP and cytokine levels of TNF-α, IL-2, IL-4 in each group are shown in Table 1. It showed that compared with sham+control-plasmid group, the serum level of VIP in OA rats which treated with control-plasmid was significantly lower, as the VIP concentrations of the two groups were 68.8 ± 9.7 pg/mL and 29.5 ± 12.4 pg/mL, respectively, whereas the VIP plasmid treatment in OA rats obviously up-regulated the VIP serum level to 57.6 ± 10.1 pg/mL. For the serum cytokine levels, it was found that the serum levels of TNF-α and IL-2 in OA + control plasmid group were significantly increased and that the serum level IL-4 was obviously decreased compared to the sham+control plasmid group and sham+VIP group (*P* < 0.05). However, in the OA rats treated with VIP plasmid, the serum cytokine levels restored to those in sham+control plasmid group or sham+VIP group, because the serum levels of TNF-α and IL-2 were markedly down-regulated, and IL-4 was significantly up-regulated (*P* < 0.05).

**Table 1:** Levels of Serum VIP and cytokines in each group ($\overline{x}$±SD, *n* = 12)

Group	VIP (pg/mL)	TNF-α (pg/mL)	IL-2 (pg/mL)	IL-4 (pg/mL)
Sham+NC plasmid	68.8 ± 9.7	106.1 ± 8.3	130.3 ± 10.5	41.2 ± 9.3
Sham+VIP plasmid	73.4 ± 11.6	101.3 ± 10.8	122.1 ± 9.9	46.7 ± 8.4
OA + NC plasmid	29.5 ± 12.4*	184.9 ± 8.6*	174.3 ± 12.7*	14.7 ± 6.6*
OA + VIP plasmid	57.6 ± 10.1^#^	112.8 ± 10.2^#^	131.4 ± 13.4^#^	43.8 ± 8.7^#^

Compared with Sham+NC plasmid group, *P < 0.05; Compared with OA + NC plasmid group, ^#^*P <* 0.05

### Identification of synoviocytes

**Fig. 2:**
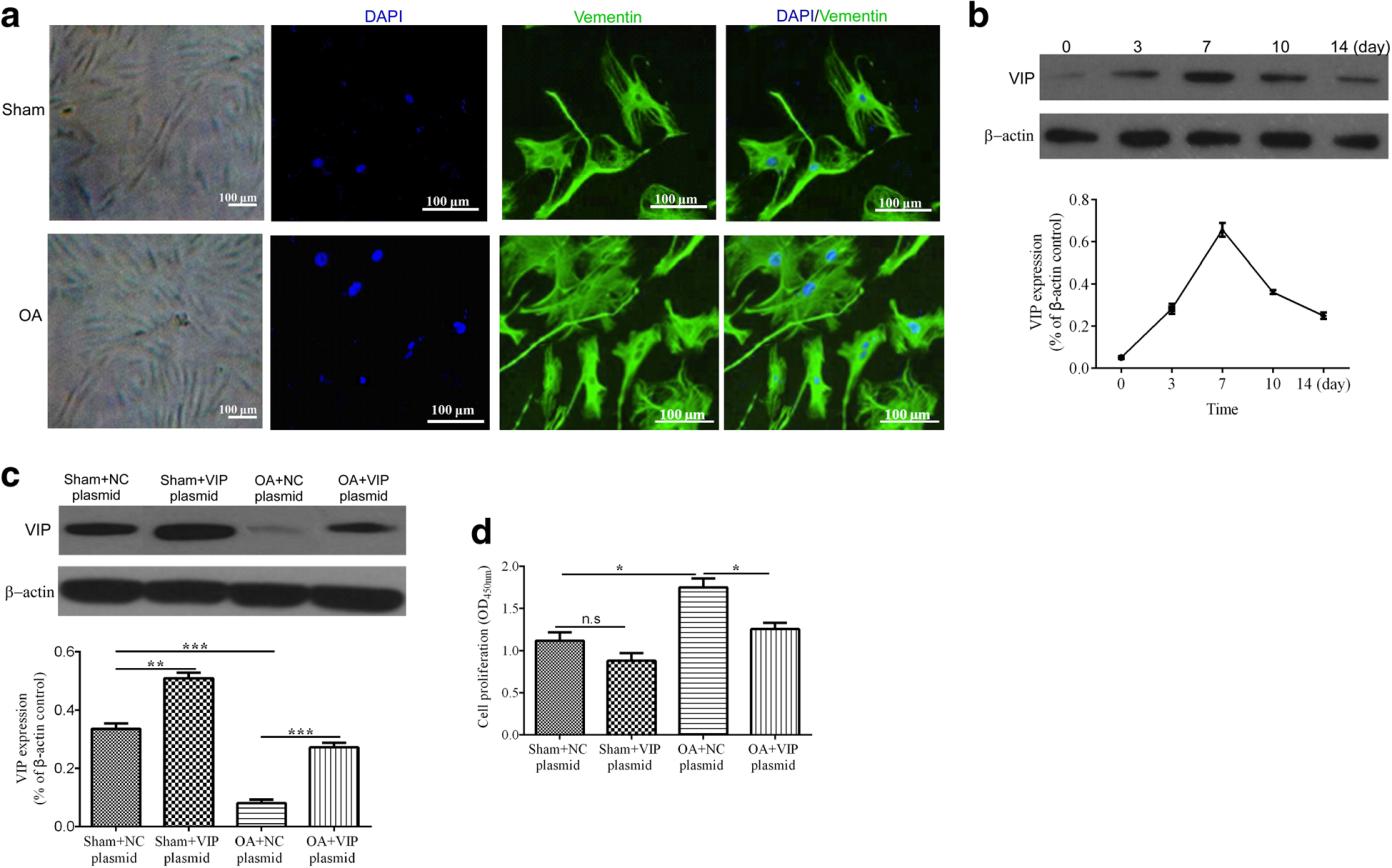
Light optical microscope and immunofluorescence staining of vimentin pictures of synoviocytes (**a**). VIP expression in OA-synoviocytes during culture for 14 days (**b**), culture for 2 days in 4 groups (**c**). VIP plasmid inhibits the proliferation of synoviocytes (**d**). *P < 0.05, **P < 0.01, ***P < 0.001, and n.s means no statistical difference

As shown in Fig. 2a, we found that the cellular morphology of the cells isolated from sham and OA model rats were all fibroblast-like. Additionally, immunofluorescence staining showed vimentin positively expressed in the cytoplasm of the two types of cells. These findings suggested that the cultured cells isolated from sham and OA rats were all fibroblast-like synoviocytes.

### Detection of transfection efficiency

To verify the transfection efficiency, the expression of VIP in transfected OA synoviocytes was detected. As shown in Fig. 2b, the level of VIP maintained at different culture time for 14 days, that the expression of VIP was gradually increased with time before the synoviocytes were cultured for 7 days. and it reached the maximum after the cells were cultured for 7 days but then declined over the next few days. Meanwhile, we also observed that the expression level of VIP in the OA synoviocytes which transfected with control-plasmid (OA + NC plasmid group) was very low, while it increased markedly in VIP plasmid transfected synoviocytes (Fig. 2c).

### VIP inhibits the proliferation of synoviocytes

After transfection for 48 h, the cell proliferation were detected. As shown in Fig. 2d, the ability of synoviocytes proliferation in OA + control plasmid group increased notably compared with sham+control plasmid group, and the difference was statistically significant (*P* < 0.01). However, in the OA + VIP plasmid group, the proliferation ability of synoviocytes decreased obviously, with levels close to the sham synoviocytes.

### Effect of VIP plasmid on the biomarkers of synoviocytes

**Fig. 3:**
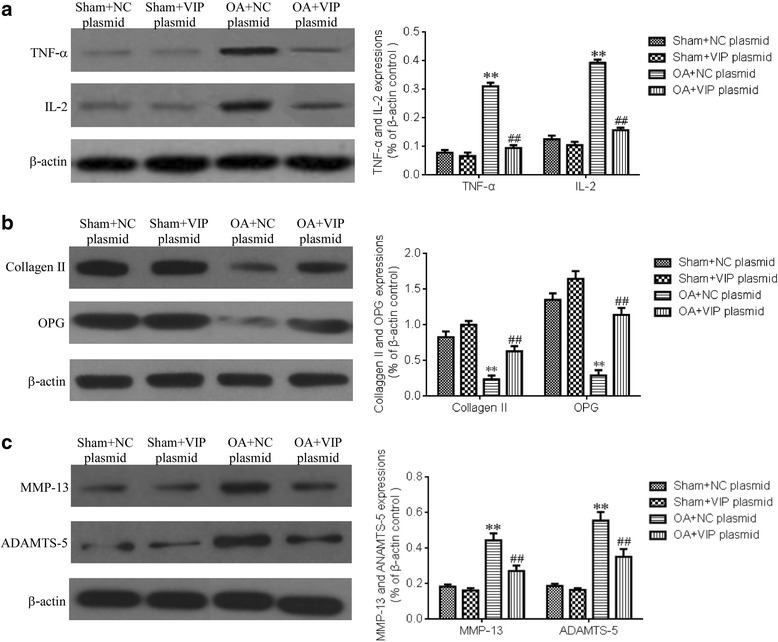
Effect of VIP plasmid on protein expressions of biomarkers in synoviocytes. TNF-α and IL-2 expressions (**a**). Collagen II and OPG expressions (**b**). MMP-13 and ADAMTS-5 expressions (**c**). Compared with Sham+NC plasmid group, ^**^P < 0.01, Compared with OA + NC plasmid group, ^##^*P* < 0.01

As is shown in Fig. 3, the effect of VIP plasmid on the protein expressions of TNF-α, IL-2, Collagen II, OPG, MMP-13 and ADAMTS-5 were displayed. Compared with the sham+control-plasmid and VIP plasmid groups, in the OA + control-plasmid group, the protein expressions of TNF-α, IL-2, MMP-13 and ADAMTS-5 were obviously increased, and the protein expressions of Collagen II and OPG in the OA synoviocytes were significantly decreased. However, in the OA synoviocytes cultured with VIP plasmid in OA + VIP plasmid group, the protein expressions of TNF-α, IL-2, MMP-13 and ADAMTS-5 were up-regulated, and the protein expressions of Collagen II and OPG were down-regulated.

### Effect of VIP plasmid on NF-κB signaling pathway

**Fig. 4:**
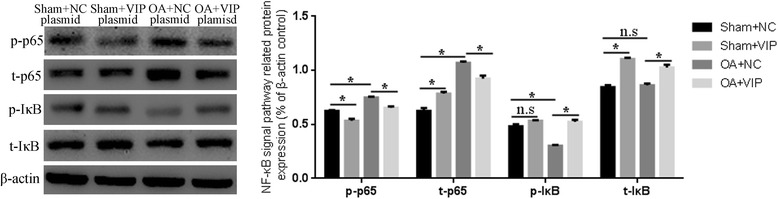
The effect of VIP plasmid on protein expression of NF-κB signaling. p-p65 and p-IκBα were the phosphorylated form of p65 and IκBα, respectively; t-p65 and t-IκBα were the total of p65 and IκBα, respectively. Left: detected via western blot; Right: relative expression

To investigate the mechanism of VIP plasmid treatment on synoviocytes, we further detected the expression of related molecular in NF-κB signal pathway, including p65 and IκBα. As is shown in Fig. 4, compared with synoviocytes of sham rats, the expression of p-p65 and p- IκBα in synoviocytes of OA rats up-regulated and down-regulated significantly, respectively (*P* < 0.05). However, after VIP plasmid transfected, the expression of p-p65 and p- IκBα in synoviocytes of OA rats decreased and increased notably compared with those in synoviocytes of OA + NC group, which indicates the treatment of VIP plasmid could effectively inhibit the NF- NF-κB signal pathway in synoviocytes.

## Discussion

Until now, in the clinical, an effective treatment strategy for OA still has been lacking. VIP, which composed of 28 amino acid residues, is a neuropeptide with anti-inflammatory and immune regulation that participates in the reconstruction of bone resorption, metabolism and fracture healing. In addition, previous researches have shown that VIP could effectively improve the symptoms of arthritis and inhibit cartilage damage [15, 16]. VIP has a short half-life and fast metabolism in vivo, and the long-term injection of VIP will produce immune suppression and gastrointestinal disorders. However, gene therapy may effectively avoid these problems. More importantly, VIP plasmid treating for OA has not been reported before in the literature. Therefore, in this study, we investigate the pathogenesis of OA and the effect of VIP plasmid on treating OA.

Firstly, we observed the effects of VIP plasmid treating for OA through establishing OA model. The results showed the pathological symptoms of OA rats were markedly improved after VIP plasmid intervention. Previously, Jiang et al. measured the VIP level of fifty patients with primary knee OA and found the VIP level in OA patients were much lower than that in heathly people, and this difference was negatively associated with progressive joint damage in OA [8]. In our study, we also found that VIP levels in OA rats were significantly lower than that in sham-operated rats. Moreover, TNF-α is an important pro-inflammatory cytokine, and can start a cascade reaction which are directly involved in the pathological changes of OA. Studies reported IL-4 has chondro protective effects, which will decrease as cartilage degraded [17]. In this study, we found that compared with the sham-operated rats, the expression of TNF-α and IL-2 increased, and IL-4 decreased in OA rats. In contrast, in rats of OA + VIP plasmid group, these inflammatory cytokines were markedly restored. These findings were consistent with a previous study that reported VIP had anti-inflammatory activity [18]. Therefore, this study suggested that to some degree, VIP plasmid has a treatment function in OA rats.

Hitherto, the pathogenesis of OA still remained unclear. A series of literature [19, 20] reported that OA was usually accompanied by an imbalance of intra-articular micro-environment, and synovial tissue and synoviocytes were reported to be the main factor, which suggested that the synovial cell microenvironment may be one of important regulatory factors in the pathogenesis of OA, and is expected to become a new target for the prevention and treatment of OA. However, the signaling pathway involved in the pathogenesis of the microenvironment of OA synoviocytes has not been reported. Synoviocytes are organizational structures that are important in maintaining the normal functions of joints and primarily consist of fibroblast-like synoviocytes and macrophage-like synoviocytes. Among those, fibroblast-like synoviocytes are not only the major component, but also the main functional cells of synovial tissue. In early OA, synovial shows hyperplasia and fibrosis, and secret inflammatory mediators, degradation enzymes and other factors to promote the degradation of cartilage cells, resulting in cell dysfunction of joint synovial, which is an important factor in the pathogenesis of OA, involving in the development of the whole pathological process [21, 22]. Thus, fibroblast-like synoviocytes are an important in vitro model for investigating the pathogenesis and drug treatment effects of OA. Therefore, in this research, we isolated and cultured fibroblast-like synoviocytes from sham-operated and OA rat in vitro, and detected VIP expression after VIP plasmid transfected into OA synoviocytes. The results showed the VIP expression in OA-synoviocytes reached its maximum levels at 7th day, then declined over the next several days. This finding also provided an evidence of the administration frequency of in vivo study. Meanwhile, Brdu assay showed VIP treatment inhibited the proliferation of synoviocytes. Additionally, compared with sham synoviocytes, the protein expressions of Collagen II and OPG were significantly downregulated, and that of TNF-α, IL-2, MMP-13 and ADAMTS-5 were obviously upregulated in OA synoviocytes treated with control plasmid. In contrast, after OA synoviocytes were treated with VIP plasmid, the protein expressions of these biomarkers were markedly restored. The result of the in vitro experiment was consistent with that of the in vivo experiment. The in vivo experiment demonstrated that the VIP plasmid could improve the pathological state and inhibit in flammatory cytokines, and the in vitro experiment showed that VIP plasmid could protect the synoviocytes, because after VIP plasmid treatment, the expressions of inflammatory cytokines and metalloproteinases in OA synoviocytes suppressed, while the expression of protective biomarkers of synoviocytes enhanced instead. In summary, VIP has the rapeutic effect on OA to some extent.

It was reported that the NF-κB signaling is a classic pathway involved in inflammatory response [23]. Therefore, further study was conducted to investigate whether OA pathogenesis and the mechanism of VIP plasmid related to NF-κB signaling pathway. A previous study reported that inflammatory cytokines, matrix-degrading enzymes and other factors could activate NF-κB signaling pathway. Once the NF-κB signaling pathway is activated, the expression of phosphorylated IκB will be rapidly up-regulated in by upstream IκB kinase and contribute to a large number of inflammatory cytokines and chemokines, such as the expression of IL-6, IL-8, adhesion molecules and matrix-degrading enzymes, resulting in regulate inflammation and cell growth state; while newly synthesized IκB could inhibit the activation of NF-κB conversely [24–26]. In this study, we found the protein expressions of p-p65 and p-IκBα in OA synoviocytes significantly up-regulated and down-regulated, respectively. But after treatment with VIP plasmid for 48 h, compared with the OA synoviocytes which treated with NC plasmid, p-p65 expression decreased and p- IκB expression increased in OA + VIP group. Thus, this study demonstrated that when OA occurred, inflammatory cytokines and matrix-degrading enzymes were increased, which activated the NF-κB signaling pathway. Then, as the NF-κB signaling pathway was activated, the secretion of the inflammatory cytokines and matrix-degrading enzymes would be promoted, and the protective biomarkers of synoviocytes would decrease. However, the phenomenon of OA could be reversed by treatment with VIP plasmid.

In conclusion, all evidences above showed the VIP recombinant plasmid could inhibit the proliferation of synoviocytes, improve the pathological symptoms of OA disease and produce a therapeutic effect on OA via NF-κB signaling pathway. This study enriched the understanding of the pathogenesis of OA, and provided a theoretical basis for the use of VIP plasmid on OA.

## Conclusion

VIP recombinant plasmid can alleviate osteoarthritis effectively via inhibiting NF-κB signaling pathway.
